# Prognostic significance of NLRP-3 expression in solid cancers: a systematic review and meta-analysis

**DOI:** 10.1080/07853890.2026.2712701

**Published:** 2026-08-20

**Authors:** Nishant Thakur, Muhammad Alamgeer, Manu Kumar, Quinton Luong, Jamshid Abdul-Ghafar, Surein Arulananda, Ashley Mansell, Daniel J. Gough

**Affiliations:** ^a^ Centre for Cancer Research, Hudson Institute of Medical Research, Clayton, VIC, Australia; ^b^ Department of Molecular and Translational Science, Monash University, Clayton, VIC, Australia; ^c^ Peninsula and South-East Oncology, Frankston, VIC, Australia; ^d^ Department of Medical Oncology, Monash Health, Clayton, Australia; ^e^ School of Clinical Sciences, Faculty of Medicine, Nursing and Health Sciences, Monash University, Clayton, Australia; ^f^ Department of Animal, Plant and Soil Sciences, La Trobe University, AgriBio, Bundoora, VIC, Australia; ^g^ Department of Pathology, Molecular and Cell-Based Medicine, Icahn School of Medicine at Mount Sinai, New York, NY, USA; ^h^ Department of Microbiology, Anatomy, Physiology and Pharmacology, La Trobe University, Bundoora, VIC, Australia

**Keywords:** NLRP-3, epithelial–mesenchymal transition, cancer stem cells, tumour microenvironment, prognosis, overall survival, and disease-free survival

## Abstract

**Background:**

The inflammasome is a critical immunological sensor comprised of NLRP-3, ASC, and CASPASE-1. Mutations in *NLRP-3* are prevalent in inflammatory diseases. However, the role of NLRP-3 in cancer is controversial. This study investigates whether NLRP-3 expression is associated with clinical outcomes in patients with solid cancers.

**Methods:**

PubMed (MEDLINE), Embase, Cochrane, and Google Scholar were searched for articles reporting NLRP-3 expression and disease outcome data in cancer patients. RevMan Review Manager was used to calculate pooled hazard ratios and Mantel–Haenszel pooled odds ratios. RNA sequencing datasets from the TCGA Pan-Cancer (PANCAN) were used for external validation.

**Results:**

Patients with higher NLRP-3 expression showed a significant association with larger tumor size, advanced tumor grade, TNM stage, and presence of metastasis. High NLRP-3 expression has a significant association with poor OS (HR:2.12, 95% CI = 1.49–3.03), *p* < 0.0001) and DFS (HR:1.86, 95% CI = 1.30– 2.65, *p* = 0.0007). Subgroup analysis showed that higher NLRP-3 expression is associated with worse OS in head and neck cancer (HR: 2.77, 95% CI = 1.88–4.09, *p* < 0.00001), colorectal cancers (HR:2.14, 95% CI= 1.59– 2.87, *p* < 0.00001), and pancreatic cancer patients (HR: 3.19, 95% CI = 1.73–5.91, *p* = 0.0002).

**Conclusion:**

High NLRP-3 expression is associated with advanced disease and poor outcomes in many solid tumours.

## Introduction

Chronic inflammation predisposes individuals to cancer development and can be attributed to 15–20% of all cancer cases [1]. Interleukin-1β (IL-1β) is a prototypic pro-inflammatory cytokine, primarily secreted by immune cells in response to inflammatory insults and is associated with poor survival in cancer patients [2,3]. The link between IL-1β and cancer was thrust into the spotlight following the Canakinumab Anti-inflammatory Thrombosis Outcomes Study (CANTOS) clinical trial of a neutralizing IL-1β antibody for atherosclerotic patients. Retrospective analysis showed that IL-1β neutralization significantly reduced the onset of lung cancer when used in the prophylactic setting [4]. IL-1β is produced in an inactive pro-form that is cleaved into its biologically active form. The Inflammasome is a primary source of biologically active IL-1β [5,6]. Inflammasomes are protein complexes typically comprised of a NOD-Like receptor (NLR) family member, with apoptosis-associated speck-like (ASC), and Caspase-1 [5,6]. The nucleotide-binding domain, leucine-rich-containing family, pyrin domain-containing-3 (NLRP-3) inflammasome complex is activated by a vast array of stimuli [7]. Typically, NLRP-3 inflammasome activation requires priming to drive expression of constituent proteins. NLRP-3 recognition of danger or pathogen-associated molecular patterns induces active inflammasome complex formation, leading to caspase-1-mediated cleavage of pro-IL1 β and pro-IL-18 into their biologically active forms [5,6]. However, mutations in inflammasome constituents, particularly *NLRP-3* that render it constitutively active cause inflammatory diseases, and cancers [8]. However, the activity of NLRP-3 appears to be a double-edged sword in cancer with reports showing tumor promotion or suppression depending on the type of cancer [5]. Given the importance of immune modulators as cancer therapies [9,10], understanding the impact of key immune regulators such as NLRP-3 is required. Therefore, in this systematic review and meta-analysis, we evaluate the association of NLRP-3 expression with patient outcomes, clinicopathological variables and cellular processes in patients with solid cancers.

## Methods

### Registration and reporting

The meta-analysis was registered with PROSPERO in 2022 (CRD42022313516), and Preferred Reporting Items for Systematic Reviews and Meta-Analyses (PRISMA) guidelines were followed [11].

### Search strategy

The electronic search was conducted in PubMed (MEDLINE), Embase, Cochrane, and Google Scholar to find articles reporting NLRP-3 expression and patient outcome data. We included studies published up to September 2024. The Medical Subject Headings (MeSH) terms and the Boolean operators (OR and AND) were combined to search for the relevant articles. The following search terminology was used: ‘The NOD-, LRR- and pyrin domain-containing protein 3 (NLRP-3) inflammasome’ OR ‘NLRP-3 inflammasome’ OR ‘NLRP-3’ OR ‘NLRP-3 prognostic significance’) AND (‘solid cancer’ OR ‘neoplasms’ OR ‘Malignant neoplasm’) AND (‘prognosis’ OR ‘survival’ OR ‘epithelial to mesenchymal transition (EMT)’ OR ‘tumor-immune infiltrate’ OR ‘immunohistochemistry (IHC)’).

### Study selection criteria

Inclusion criteria [1]: studies assessing the NLRP-3 expression in post-surgical solid cancer patients through IHC [2], a comparison of prognostic endpoints including overall survival (OS), disease-free survival (DFS), and progression-free survival (PFS) with NLRP-3 expression based on IHC staining (high vs low) [3], studies showing the relationship between NLRP-3 expression based on IHC staining (high vs low) and clinicopathological variables and/or markers of biological process. High and low NLRP-3 expression was determined by scoring IHC staining including receiver operating characteristic (ROC) curve cutoffs (cutoff range from 0 to 80%) and Immunoreactivity Score (IRS) (score range from 0 to 12). Exclusion criteria [1]: duplicate reports, meta-analysis, reviews, conference abstracts, ongoing investigations, news reports, epidemiological studies, autopsy series, non-English articles [2]; articles whose experimental design and research methodologies were performed exclusively on cell lines or animal models [3]; articles with Newcastle–Ottawa quality assessment scale (NOS) scores of less than 6. The QUIPS tool was also used to evaluate the risk of bias in the included studies [12]. Grades of Recommendation, Assessment, Development, and Evaluation (GRADE) was used to evaluate the certainty of evidence for OS and DFS [13].

### Data extraction and collection

NT and DJG independently retrieved patient data from the included studies and organized data in the Review Manager (RevMan) software [14]. Any conflicts were resolved *via* consensus between the reviewers MA, SA, MK, QL, and AM, who participated in the process. Subsequently, we collected the main study features, including first author, year of publication, country, ethnicity, cancer subtype, clinical stage, tumor grade, number of patients, median age, follow-up period, chemotherapy regimen, NLRP-3 detection method, hazard ratio (HRs) and 95% confidence interval (CI) for OS and D/PFS. If the HR value was missing, we contacted the corresponding author for the information. If not provided, NT independently used a web plot digitizer to extract the data from Kaplan–Meier curves [15] and calculated the HR using the methods published by Parmar et al. [16].

### Quality assessment and publication bias

DJG and NT independently assessed the quality of studies. We used NOS to evaluate the quality of studies, including three domains: selection, comparability, and outcomes assessment, with a score of 0–9. Scores >6 represent high quality and were included in this study. To assess the risk of bias across the studies, the QUIPS tool was used, using six domains: study participation, study attrition, prognostic factor measurement, outcome assessment, study confounding, and statistical analysis and reporting. Each domain was reported as low, moderate and high risk of bias. If any discrepancies were highlighted, then JAG and AM participated in the process and resolved them by consensus. We calculate the Cohen’s kappa coefficient (κ) to evaluate the interobserver agreement. A *κ* value higher than 0.70 is considered a good agreement, 0.40–0.75 is considered a moderate agreement, and <0.40 is considered a poor agreement. Further, NT independently used GRADE to assess the overall certainty of the evidence based on five factors: risk of bias, indirectness, inconsistency, imprecision, and publication bias. Based on the judgment, the overall certainty of the evidence was classified as high, moderate, or low.

The symmetry of Funnel plots and Begg’s and Egger’s regression tests was used to assess publication bias. If potential publication bias existed, the trim and fill methods were performed to investigate the cause [9]. NT and DJG performed subgroup, meta-regression, and sensitivity analysis to investigate the source of heterogeneity in the included studies.

### Validation analysis

Transcriptomic data from The Cancer Genome Atlas (TCGA) Pan-Cancer (PANCAN) cohorts were analyzed using the UCSA Xena platform to validate the association between *NLRP-3* expression and prognosis [10]. High *NLRP-3* is defined as greater than the mean and Low *NLRP-3* expression is lower than mean of *NLRP-3* expression across the entire cohort.

### Statistical analysis

RevMan (version 5.3; Cochrane Collaboration, Copenhagen) [14] was used to calculate all pooled HRs and 95% Confidence intervals (CIs) for risk of mortality and DFS in NLRP-3 (high vs low expression) patients. We combined the HR and 95% CI of individual studies to obtain pooled HR. Conventionally, a pooled HR of more than 1 demonstrates poor survival outcomes with higher NLRP-3 expression. *I*^2^ indicates the heterogeneity; a score of 0–25% represents low heterogeneity, 26–75% represents moderate heterogeneity, and 76–100% represents substantial heterogeneity. The DerSimonian–Laird random model was used for all forest maps due to the moderate heterogeneity observed in all studies (*I*^2^ = 75%). RevMan software[14] was used to perform overall pooled, subgroup, and sensitivity analysis, while Comprehensive Meta-analysis Software (V4) [17] were used to perform t meta-regression, and publication bias analysis. Mantel–Haenszel pooled odds ratios (ORs) with 95% CIs were used to determine the relationship between NLRP-3 expression and clinical variables. A *p* value of less than 0.05 was considered statistically significant.

## Results

### Characteristics and quality assessment of included studies

**Figure 1.**
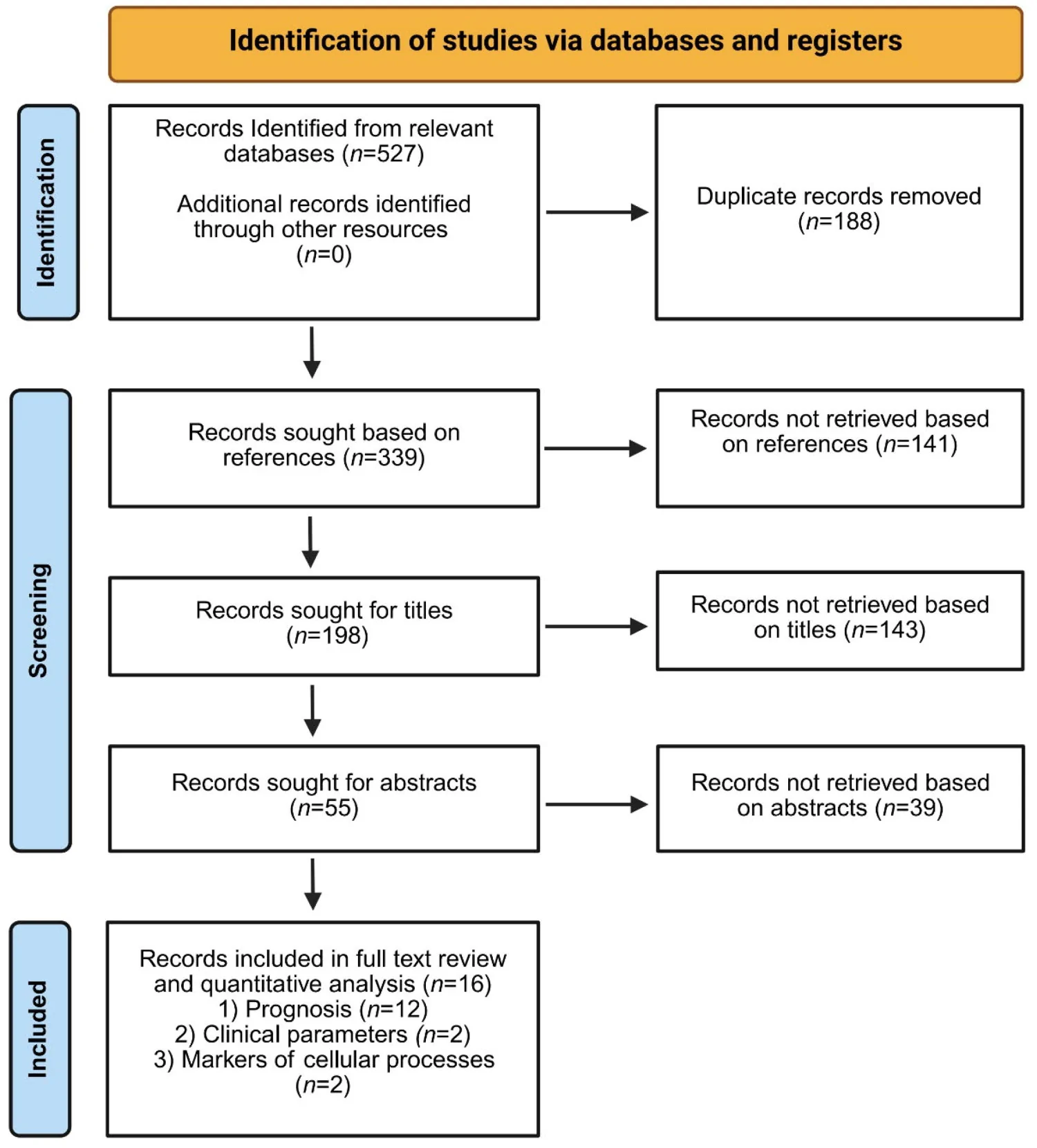
Prisma diagram of the screening process of included studies.

A primary literature search identified 527 records published prior to September 2024. Sixteen records met the inclusion criteria and were eligible for full-text review [18–30]. Prognosis-based studies (*n* = 12), clinical parameter studies (*n* = 2), and studies reporting markers of cellular processes (*n* = 2) were identified (Figure 1). Twelve records with a total of 1,731 patients were eligible for this meta-analysis [18–22,24–27,29]. Figure 1 summarizes the identification and screening process and includes records based on PRISMA guidelines.

Geographically these studies were performed in Asia ((China (*n* = 9) [18–24,26,31] and South Korea (*n* = 1) [29]) and Europe ((Italy (*n* = 1) [25] and Spain (*n* = 1)) [27]. They include seven solid tumor types: colorectal cancer (CRC) (*n* = 3) [22–24], endometrial cancer (EC) (*n* = 1) [29], head and neck squamous cell carcinoma (HNSCC) (*n* = 3) [18,20,26], non-small lung cancer (NSCLC) (*n* = 1) [19], osteosarcoma (*n* = 1) [31], breast cancer (BC) (*n* = 1) [25] and pancreatic ductal carcinoma (PDAC) (*n* = 2) [21,27] (Table 1).

**Table 1.** The main features of the included studies in this meta-analysis.

Author/year	Country	Ethnicity	Cancer (Clinical stage)	Tumour grade	Patients number	Median age (median range)	Detection method	Hazard ratio CI 95%	NOS score
Feng/2017 [20]	China	Asian	OSCC* (NA)	I–III	103	NA	IHC	OS: 3.81(0.36–8.08) DFS: 1.88 (0.33–4.12)	7
Xue/2019 [18]	China	Asian	LSCC* (I–IV)	I–III	104	NA	IHC	OS: 1.94 (1.026–3.667)	7
He/2020 [19]	China	Asian	NSLC* (I–IV)	I–III	144	NA	IHC	OS: 1.080 (0.947–1.231)	7
Shao/2020 [22]	China	Asian	CRC* (I–IV)	I, III	69	NA	IHC	OS: 1.89 (1.29–2.76)	7
Wang/2020 [23]	China	Asian	CRC* (I–IV)	I, III	135	NA	IHC	OS: 2.14 (1.32–3.47)	7
Huang/2021 [31]	China	Asian	OS# (I, IIA, IIB)	NA	55	NA	IHC	OS: 4.09 (1.29–12.97) DFS: 2.92 (1.40–6.05)	7
Saponaro/2021 [25]	Italy	Caucasian	BC* (NA)	I–III	374	53	IHC	OS: 0.79 (0.30–2.09 DFS: 1.75 (0.98–3.15)	8
Shi/2021 [24]	China	Asian	CRC* (I–IV)	I–III	100	62.4	IHC	OS: 3.11 (1.20–8.09)	7
Chen/2022 [26]	China	Asian	HNSCC* (NA)	I–III	157	NA	IHC	OS: 3.14 (1.67–5.92)	7
Zheng/2022 [21]	China	Asian	PDAC* (I–II)	I–III	98	58.4	IHC	OS: 3.11 (1.19–8.13)	7
Chan Ha/2024 [29]	South Korea	Asian	EC$ (I–IV)	I–III	351	54.1	IHC	OS:1.091 (0.383–3.108) DFS:1.17 (0.514–2.691)	8
Fraile-Martinez/2024 [27]	Spain	Caucasian	PDAC* (I, III, IV)	NA	41	72	IHC	OS:2.06 (1.34–3.17)	6

Abbreviation: NSC: nasopharyngeal carcinoma, OSCC: oral squamous cell carcinoma, LSCC: laryngeal squamous cell carcinoma. NSLC: non-small lung cancer, OS: osteosarcoma, BC: breast Cancer, NPC: nasopharyngeal carcinoma, HNSCC: head and neck squamous cell carcinoma, CRC: colorectal cancer, PDAC: pancreatic ductal adenocarcinoma, DCF: Docetaxel, cisplatin, and 5-fluorouracil, EC: endometrial cancer, NA: not available, IHC: immunohistochemistry, OS: overall survival, DFS: disease-free survival, *: TNM staging, #: Enneking staging, $: FIGO staging.

Sample sizes ranged between 55 and 374, and the median age ranged between 37 and 84 years (Table 1). Eleven studies reported a correlation between NLRP-3 and OS and four studies reported a correlation with DFS (Table 1; Supplementary Table 1). The most common method used for sampling was whole tissue sections (*n* = 7), and the immunoreactivity score (IRS) (*n* = 9) with cutoff values for IHC-based expression results differing between studies (Supplementary Tables 1 and 2). Antibodies used in IHC were from Abcam (*n* = 3), Proteintech (*n* = 2), and Sigma-Aldrich (Supplementary Table 2).

**Table 2.** Subgroup analysis between NLRP-3 expression and OS according to ethnicity, treatment regimen, cancer-specific, chemotherapy-based, analysis type, sample size, sampling format, source of HR extraction, antibody clones, and IHC scoring method.

Variables	Number of studies	Number of patients	HR (95% CI)	*p*-value	Heterogeneity		
					*I*^2^ *(%)*	*p-*value	Model
**Ethnicity**							
Asian	10	1,316	2.15 [1.48–3.12]	<0.0001*	78	<0.00001*	Random
Caucasian	2	415	1.89 [0.35–10.21]	0.46	85	0.01	Random
**Treatment regimen**							
Chemotherapy	2	158	3.89 [2.07–7.31]	<0.0001*	0	0.92	Random
Non-chemotherapy	10	1573	1.92 [1.34–2.76]	0.0004*	77	<0.0001*	Random
**Cancer types**							
Head and neck cancer	3	364	2.77 [1.88–4.09]	<0.00001*	2	0.36	Random
Colorectal cancer	3	304	2.14 [1.59–2.87]	<0.00001*	0	0.70	Random
Pancreatic cancer	2	139	3.19 [1.73–5.91]	0.0002*	0	0.32	Random
Miscellaneous cancers	4	924	1.23 [0.75–2.04]	0.41	45	0.14	Random
**Analysis types**							
Univariate	5	1,108	1.56 [0.96–2.53]	0.07	67	0.02	Random
Multivariate	8	1,172	2.10 [1.32–3.33]	0.002*	81	<0.00001*	Random
**Sample size**							
<100	4	263	2.51 [1.72–3.67]	<0.00001*	13	0.33	Random
>100	5	1,468	1.87 [1.21–2.88]	0.005*	78	<0.0001*	Random
**Sampling format**							
Tissue microarray	5	1,073	1.35 [0.90–2.04]	<0.15	50	0.09	Random
Whole tissue section	7	658	2.54 [1.49–3.22]	<0.00001*	0	0.47	Random
**Source of HR extraction**							
Directly	9	1,468	1.98 [1.29–3.04]	0.002*	78	<0.0001*	Random
Indirectly	3	263	2.41 [1.57–3.70]	<0.0001*	22	0.28	Random
**Antibody types**							
Abcam	3	214	2.27 [1.52–3.41]	<0.0001*	28	0.25	Random
Proteintech	2	244	1.64 [0.60–4.52]	0.34	78	0.03	Random
Sigma Aldrich	2	260	3.40 [2.10–5.52]	<0.00001*	0	0.70	Random
**Antibody clones**							
Monoclonal antibody	1	351	1.09 [0.38–3.11]	0.87	NA	NA	Random
Polyclonal antibody	9	849	2.28 [1.57–3.31]	<0.0001*	81	<0.00001*	Random
**IHC scoring methods**							
IRS	11	1357	2.28 [1.57–3.31]	<0.0001*	79	<0.00001*	Random
ROC	1	374	0.79 [0.30–2.09]	0.63	NA	NA	Random

HR: hazard ratio, CI: confidence interval, IRS: Immunoreactivity score, ROC: receiver operating characteristic curve, NA: not available.

*Significant *p* value.

### Quality of control, risk of bias, and certainty of evidence analysis

The overall quality of included studies was assessed using the NOS score. Of these studies, 11 (68.75%) achieved a score of 7 or higher, indicating high-quality study design (Table 1; Supplementary Table 3). The QUIPS method was employed to assess bias risk across the 16 studies. Among them, six studies (37.5%) were classified as having a high risk of bias, eight studies (50%) had a moderate risk, and two studies (12.5%) had a low risk (Supplementary Table 4). The inter-rater agreement between the two reviewers was excellent for both NOS and QUIPS, with Cohen’s κ = 1.00 (Supplementary Table 5). Next, we applied the GRADE approach to evaluate the certainty of evidence for studies reporting OS and DFS. The OS was rated very low due to risk of bias, inconsistency, and publication bias. Similarly, DFS was rated very low because of risk of bias, indirectness, and imprecision (Supplementary Table 6).

### Correlation between NLRP-3 expression, overall, and disease-free survival

**Figure 2.**
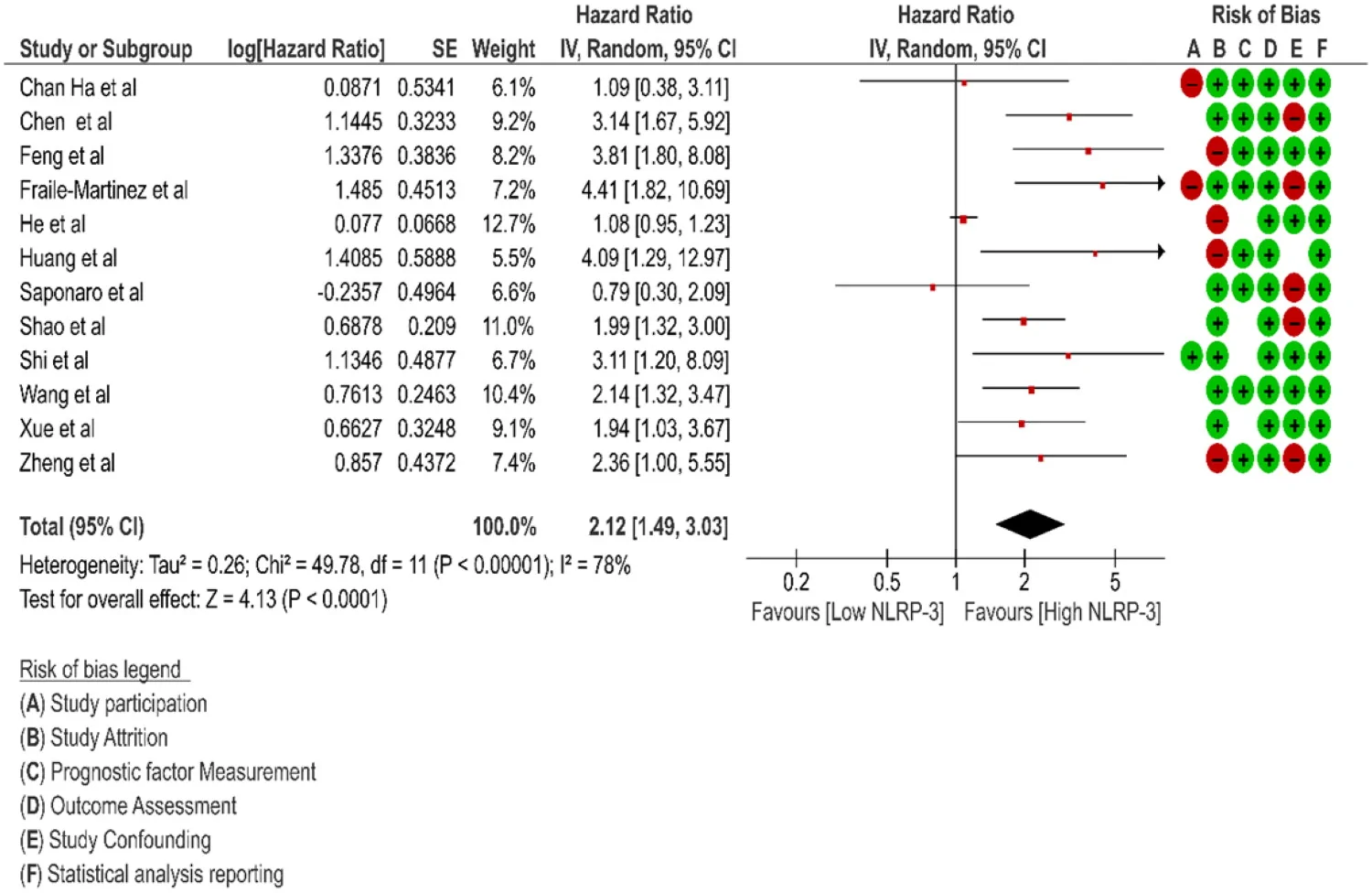
Forest plot for the relationship between NLRP-3 expression and overall survival. The hazard ratio (HR) and the corresponding 95% confidence interval (CI) were also reported for each study. A hazard ratio of more than 1 is associated with a poor prognosis, and a ratio below 1 is considered a good prognosis.

Twelve studies with 1,731 patients reported OS, and pooled results revealed that higher expression of NLRP-3 was significantly associated with poor OS (hazard ratio (HR): 2.12, 95% confidence interval (CI):1.49–3.03, *p* < 0.0001) (Figure 2). Since the heterogeneity was high (*I*^2^ = 78%), subgroup analysis was conducted to investigate the source of heterogeneity (Table 2; Supplementary Figure 1). This revealed that Asian patients with higher NLRP-3 expression had significantly inferior survival outcomes (HR: 2.15, 95% CI: 1.48, 3.12, *p* < 0.00001*) (Table 2; Supplementary Figure 1). NLRP-3 expression did not show any correlation with outcome in Caucasian patients. However, 83% of studies were from Asian populations and Caucasians made up 24% of the total cases analyzed. Therefore, additional studies to balance ethnicity in analyses would be required to confirm this observation.

Patients with higher NLRP-3, who received chemotherapy (HR: 3.89, 95% CI: 2.07, 7.31, *p* < 0.0001*), had significantly worse OS compared to patients with no chemotherapy treatment (HR: 1.92, 95% CI: 1.34, 2.76, *p* = 0.0004*) (Table 2; Supplementary Figure 1). Subgroup analysis showed that patients with higher NLRP-3 expression had significantly worse OS across cancer subtypes, including (HNSCC) (HR: 2.77, 95% CI:1.88–4.09, *p* < 0.00001), (CRC) (HR: 2.14, 95% CI:1.59–2.87, *p* < 0.00001), and (PDAC) (HR: 3.19, 95% CI: 1.73–5.91, *p* = 0.0002) (Table 2; Supplementary Figure 1). Intriguingly, an opposing trend was observed for hepatocellular carcinoma (HCC) [28]. In this tumour type, NLRP-3 expression was observed to be elevated in tumour-adjacent normal tissue [32] ,and lower NLRP-3 expression was associated with advanced clinicopathological features [28]. We combined 924 patients with osteosarcoma, endometrial, breast, and lung cancers into a single group, hereafter referred to as miscellaneous cancers. However, no significant difference between NLRP-3 and outcome was observed (HR: 1.23 (CI 0.75, 2.04), *p* = 0.14) (Table 2; Supplementary Figure 1). This could be due to the different types of cancers included in this group and therefore, results should be interpreted cautiously (Table 2; Supplementary Figure 1).

**Figure 3.**
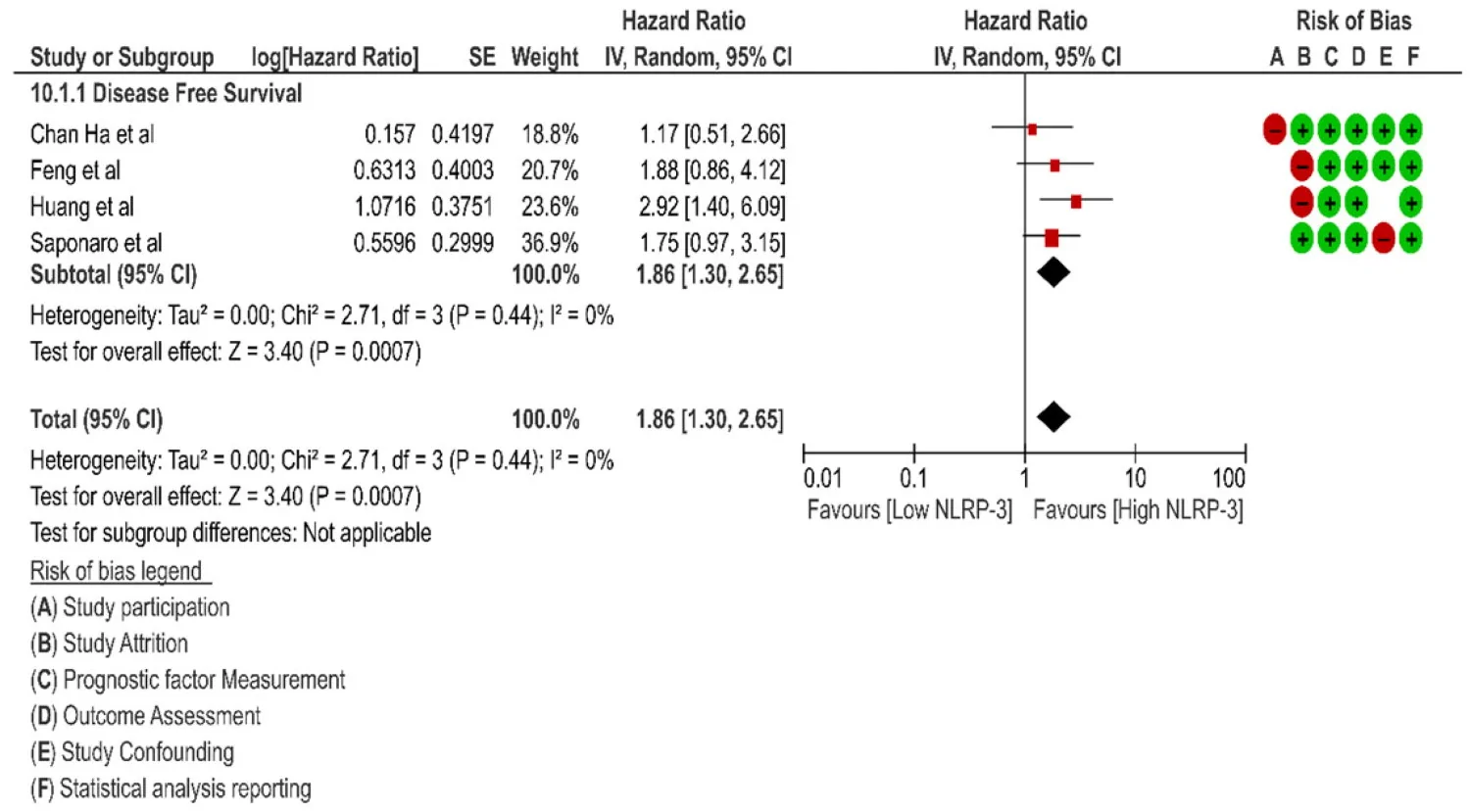
Forest plot for the relationship between NLRP-3 expression and disease-free survival. The hazard ratio (HR) and the corresponding 95% confidence interval (CI) were also reported for each study. A hazard ratio of more than 1 is associated with a poor prognosis, and a ratio below 1 is considered a good prognosis.

We confirmed the inferior prognostic impact of higher NLRP-3 expression with multivariate analysis methods (HR: 2.10, 95% CI: 1.32–3.33, *p* < 0.002), sample size <100 (HR: 2.51, 95% CI:1.72–3.67, *p* < 0.00001) vs. >100 (HR: 1.87, 95% CI:1.21–2.88, *p* < 0.005), whole tissue sampling method (HR: 2.54 (95% CI:2.01–3.22, *p* < 0.00001), and dircet HR extraction (HR: 1.98, 95% CI:1.29–3.04, p = 0.002) and indirect source extraction (HR: 2.41, 95% CI:1.57–3.70, p < 0.0001) (Table 2; Supplementary Figures 1 and 2). This demonstrated inferior OS in patients with higher NLRP-3 expression (Table 2; Supplementary Figure 1). Based on antibody type, Abcam (HR: 2.27, 95% CI: 1.52–3.41, *p* < 0.0001) and Sigma-Aldrich (HR: 3.40, 95% CI:2.10–5.52, *p* < 0.00001) were significantly associated with poor OS outcome in patients with high NLRP-3 expression (Table 2; Supplementary Figure 2). According to the antibody clones, polyclonal antibody (HR: 2.33, 95% 1.54–3.51, *p* < 0.0001) and IRS IHC evaluation methods or scoring system (HR: 2.28, 95% 1.57–3.31, *p* < 0.0001) showed a significant association with inferior OS outcome in patients with higher NLRP-3 expressions (Table 2; Supplementary Figure 2). Similarly, four studies (583 patients) showed that higher NLRP-3 expression was significantly associated with inferior DFS (HR: 1.86, 95% 1.30–2.65, *p* = 0.0007) (Figure 3).

### Meta-regression analysis

To determine whether antibody clones, IHC evaluation methods (IRS vs ROC), and sampling method (WTS vs TMA) contributed to heterogeneity, we performed univariate regression analysis. This revealed that IHC evaluation methods (*p* = 0.020 and *R*^2^ =1.00) and sampling methods (*p* = 0.0010 and *R*^2^ = 0.73) were the primary source of heterogeneity (Table 3; Supplementary Figure 3). Standardised IHC evaluation methods and sampling methods to evaluate the NLRP-3 expression are recommended for future studies.

**Table 3.** Univariate meta-regression analysis of factors contributing to heterogeneity.

Variables	No of studies	Coefficient (β)	Standard error	95%CI	*p* value	*R* ^2^
Antibody clones (mAb vs pAb)	10	0.721	0.693	(−0.63-2.08)	0.298	0.00
IHC evaluation methods (IRS vs ROC)	10	1.184	0.511	(0.18–2.18)	0.020*	1.00
Sampling methods (WTS vs TMA)	12	0.729	0.222	(0.29–1.16)	0.0010*	0.73

CI: confidence interval, mAb: monoclonal Antibody, pAB: polyclonal antibody, WTS: whole tissue section, TMA: tissue microarray, IRS: immunoreactivity score, ROC: receiver operating characteristic curve.

*Significant *p* value.

### Sensitivity analysis

**Figure 4.**
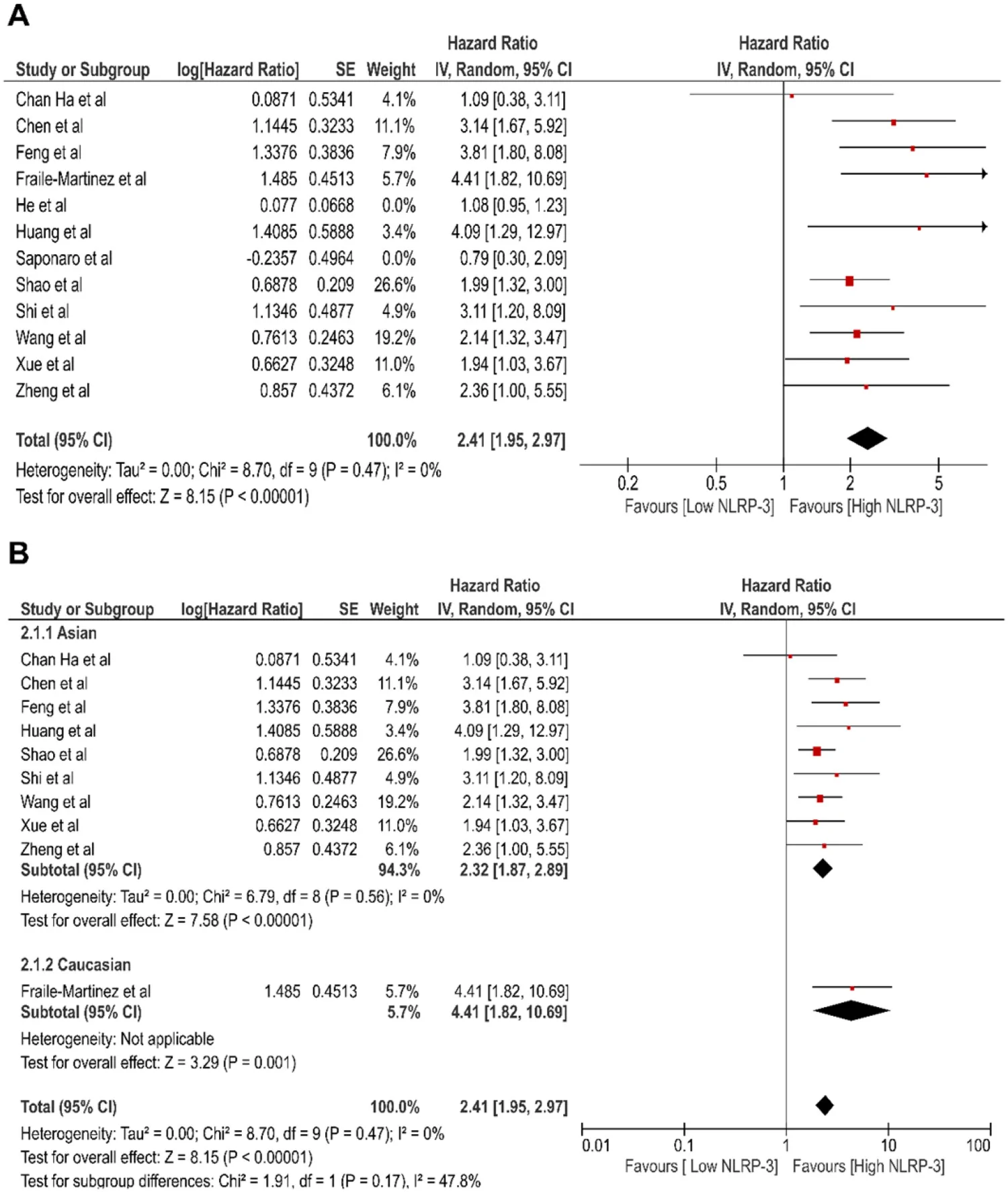
Sensitivity analysis of the association of NLRP-3 expression with survival outcome, including all studies (A), classified based on ethnicity (B). The hazard ratio (HR) and the corresponding 95% confidence interval (CI) were also reported for each study. A hazard ratio of more than 1 is associated with a poor prognosis, and a ratio below 1 is considered a good prognosis.

Sensitivity analyses were conducted to examine the robustness of this analysis. Notably, the exclusion of individual studies did not change the pooled OS, suggesting the overall prognostic value remained consistent throughout the analysis. However, we observed that He et al. [19] contributed the highest heterogeneity (*I^2^ =* 26%), followed by Saponaro et al. [25], (*I^2^ =* 0%). Therefore, we repeated the analysis excluding these studies. The recalculated outcome still showed a significant relationship between high NLRP-3 expression and worse outcomes (HR = 2.41, 95% CI: 1.95–2.97, *p* < 0.00001, *I^2^* = 0%) (Figure 4A), supporting the overall conclusions. Further, ethnicity-wise sensitivity analysis was performed. In the Asian subgroup (*n* = 10), He et al. [19] were the primary contributors, causing significantly higher heterogeneity (*I^2^ =* 78%). Excluding this study reduced heterogeneity to 0%, and the overall conclusion of NLRP-3 associated with inferior OS remained consistent with the remaining studies (HR = 2.32, 95% CI: 1.87–2.89, *p* < 0.00001, *I^2^*=0%) (Figure 4B). In the Caucasian subgroup (*n* = 2) substantial heterogeneity was observed (*I^2^=*85%). Sensitivity analysis showed that the exclusion of Saponaro et al. [25] reduced heterogeneity to 0%. However, only one study remained after the exclusion, and a meta-analysis could not be performed (Figure 4B). Together, this data showed that the result of the NLRP-3 outcome in Caucasians should be interpreted with caution because of limited studies and higher heterogeneity.

### Publication bias

**Figure 5.**
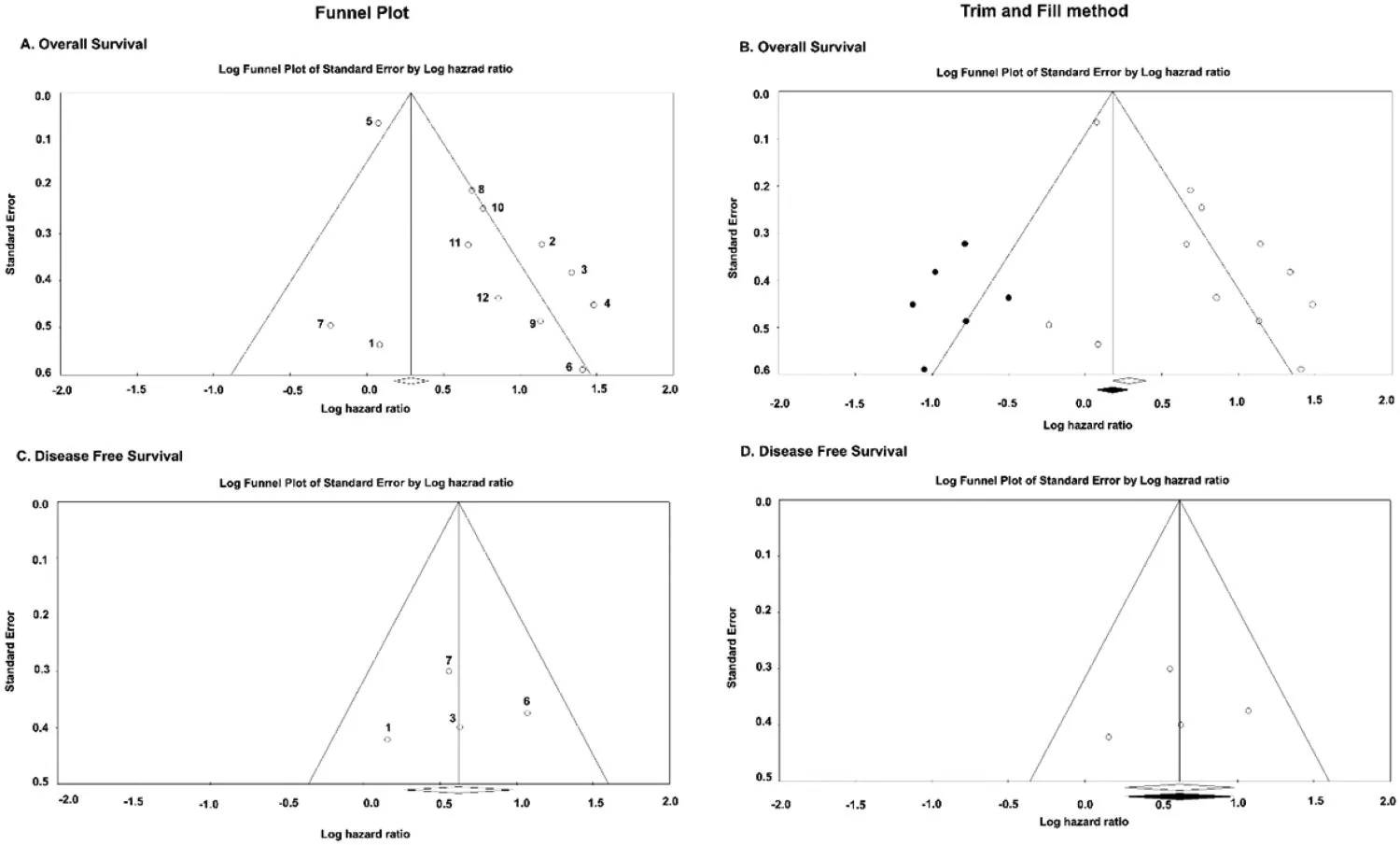
Funnel and trim-and- fill funnel plot for overall survival (A and B) and disease-free survival (C and D). 1: Chan Ha et al.; 2: Chen et al.; 3: Feng et al.; 4: Fraile-Martinez et al.; 5: He et al.; 6: Huang et al.; 7: Saponaro et al.; 8: Shao et al.; 9: Shi et al.; 10: Wang et al.; 11: Xue et al.; 12 Zheng et al.

Funnel plots showed asymmetry; therefore, we used the trim and fill method to account for this (Figure 5). Begg’s test did not show publication bias in OS and DFS data (Table 4). In contrast, Egger’s test identified publication bias in OS (*p* = 0.002), but not in DFS (Table 4).

**Table 4.** Publication bias for overall survival and disease-free survival.

Parameters	Number of studies	Publication bias (*p* value)		
		Begg’s test	Egger’s test	Model
Overall survival	12	0.83	0.002*	Random
Disease-free survival	4	0.73	0.85	Random

*Significant *p* value.

### External validation of NLRP-3 prognostic significance with Pan-cancer TCGA dataset

**Figure 6.**
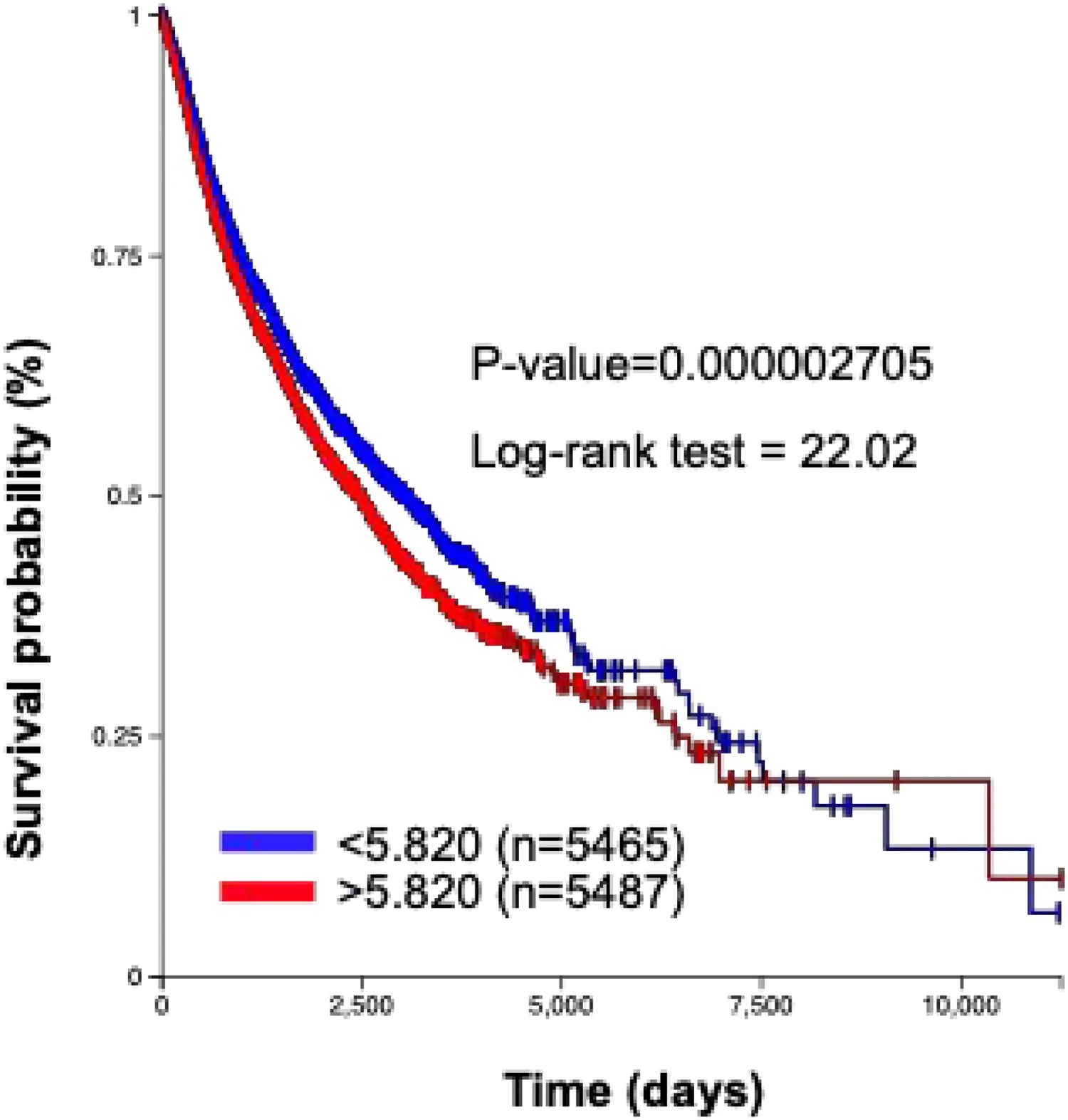
**External validation of *NLRP3* expression and overall survival analysis in the Cancer Genome Atlas (TCGA) PANCAN patients dataset.** Kaplan–Meier survival curves demonstrate the association of high (red color) vs. low (blue color) *NLRP3* mean expression in TCGA pan cancer dataset using UCSC Xena platform (*n* = 10,952).

To further independently validate the association between NLRP-3 and survival outcomes in cancer patients. We utilized the UCSC Xena browser to analyze publicly available pan-cancer RNA-expression data from the Cancer Genome Atlas (TCGA). Patients were stratified into two groups based on *NLRP-3* expression: high (greater than the mean) and low expression (lower than the mean). Notably, high *NLRP-3* gene expression was significantly associated with worse OS (*p* = 2.7e–6) (*n* = 10,952) (Figure 6).

### Relationship between clinicopathological parameters and NLRP-3 expression

We observed that patients with higher NLRP-3 expression were significantly associated with larger tumor size (>5 cm) (pooled odds ratio (OR): 1.86, 95% CI: 1.05–3.29, *p* = 0.03), advanced tumor grade (grade III) (OR: 1.52, 95% CI: 1.06–2.17, *p* = 0.02), more advanced tumor stage (III and IV stage) (OR: 4.13, 95% CI: 2.29–7.45, *p* < 0.00001), presence of lymph node metastasis (OR: 2.99, 95% CI:1.50–5.97, *p* < 0.002), distant metastasis (OR: 4.01, 95% CI: 1.67–9.68, *p* = 0.002), presence of venous (OR: 3.64, 95% CI: 1.73–7.67, *p* < 0.0007) or neural invasion (OR: 3.51, 95% CI: 1.88–6.53, *p* < 0.0001) (Table 5; Supplementary Figures 4–6).

**Table 5.** The pooled odds ratio with a corresponding 95% confidence interval between NLRP-3 expression and various clinicopathological parameters.

Parameters	Number of studies	Number of patients	Pooled odds ratio (95% CI)	*p* value	Heterogeneity		
					*I*^2^ (%)	*p* value	Model
Age (>60 vs <60)	4	411	1.14 (0.75–1.71)	0.54	0	0.71	Random
Gender (female vs male)	6	606	1.06 (0.76–1.49)	0.73	0	0.74	Random
Tumor size (>5 cm vs <5 cm)	2	204	1.86 (1.05–3.29)	0.03*	0	0.37	Random
Tumor grade (I + II vs III)	4	662	1.52 (1.06–2.17)	0.02*	6	0.36	Random
Tumor stage (I + II vs III+IV)	5	551	4.13 (2.29–7.45)	<0.00001*	23	0.27	Random
Lymph node metastasis (present vs absent)	9	1330	2.99 (1.50–5.97)	<0.002*	84	<0.00001	Random
Distant metastasis (present vs absent)	3	258	4.01 (1.67–9.68)	0.002*	0	0.79	Random
TNM stage (I + II vs III+IV)	5	552	5.70 (3.11–10.44)	<0.00001*	55	0.06	Random
Venous invasion (present vs absent)	2	204	3.64 (1.73–7.67)	<0.0007*	23	0.25	Random
Neural invasion (present vs absent)	2	204	3.51 (1.88–6.53)	<0.0001*	0	0.39	Random
Alcohol (never vs ever)	3	351	1.44 (0.91–2.28)	0.12	0	0.66	Random
Smoking (never vs ever)	3	351	1.11 (0.72–1.73)	0.65	8%	0.34	Random

* Significant *p* value.

### Association between NLRP-3 expression and markers of tumor-associated cellular processes

**Figure 7.**
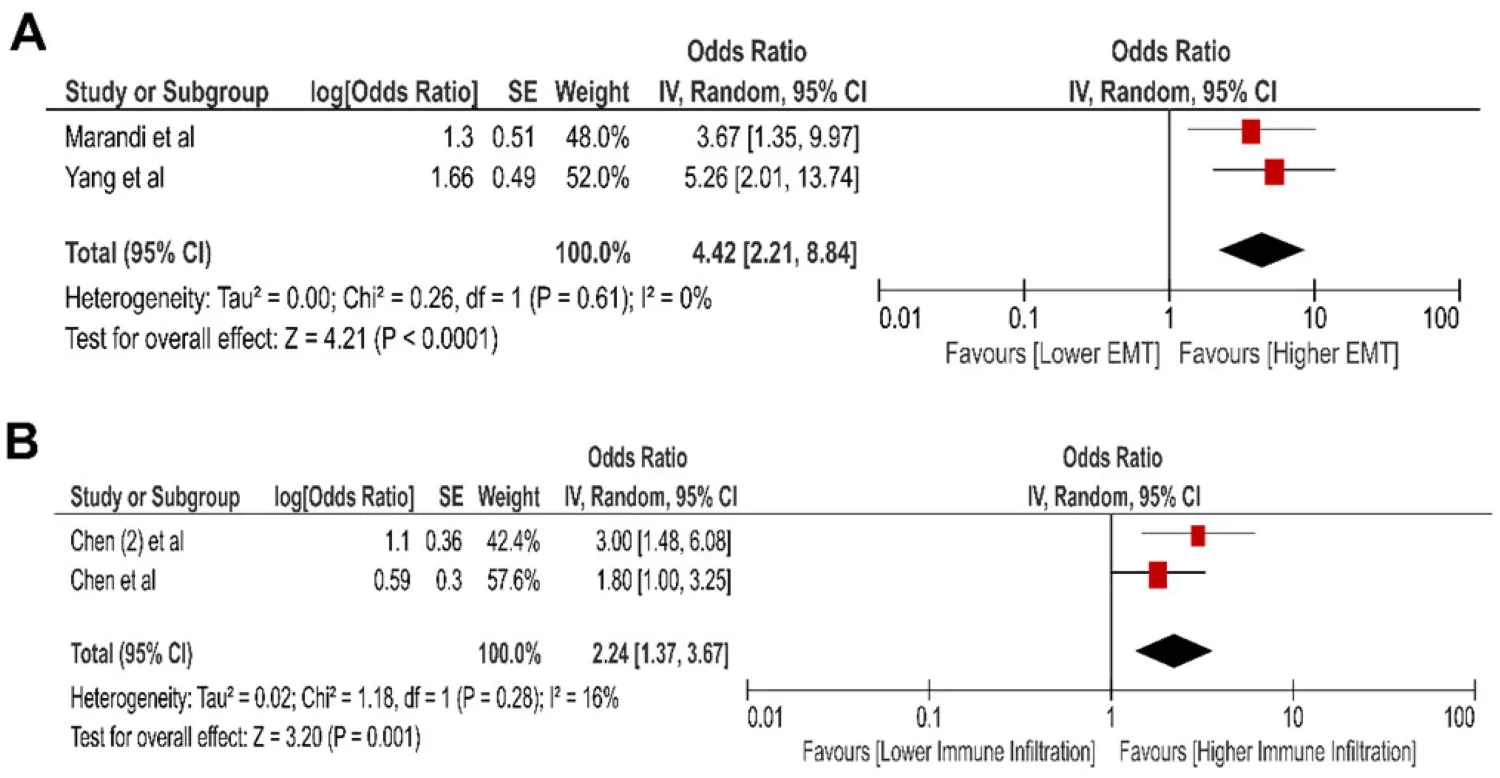
Forest plot showing the relationship between NLRP-3 expression and epithelial-to-mesenchymal transition (EMT) (A) and immune cell infiltration (B). Higher EMT indicates higher expression of Vimentin, and higher expression of tumour-associated macrophages (TAMs) indicates higher expression of CD163. A value greater than 1 reflects higher EMT and TAMs. The odds ratio, along with the corresponding 95% confidence interval (CI), was also reported for each study.

**Figure 8.**
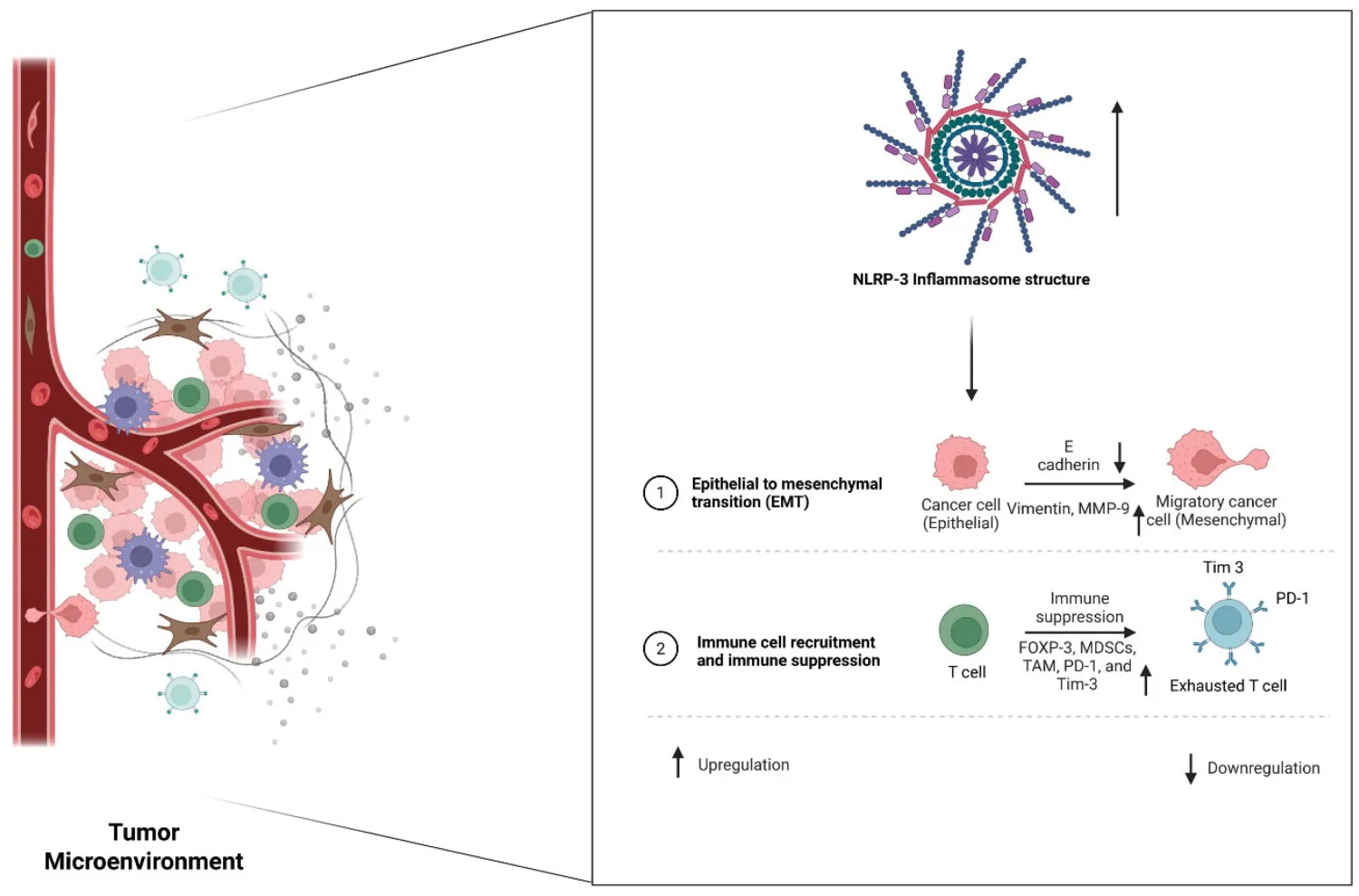
Higher expressions of NLRP-3 regulate epithelial-to-mesenchymal (EMT) and immune suppression in the tumor microenvironment, contributing to the cancer’s progression. 1) Activation of NLRP-3 promotes the epithelial-to-mesenchymal transition by increasing vimentin and matrix metalloproteinase (MMP-9) expression and downregulating E-cadherin expression, which increases invasiveness and cellular motility of cancerous cells. Simultaneously, NLR-3 promotes the immunosuppressive environment by increasing the expression of immunosuppressive markers such as tumor-associated macrophages (TAM), forkhead box protein 3 (FOXP3), myeloid-derived suppressor cells (MDSCs), T cell immunoglobulin mucin-3 (Tim3), and programmed death-1 (PD-1). The arrow indicates the direction of specific marker expression, with upregulation (↑) and downregulation (↓).

Poor clinical outcomes associated with high NLRP-3 expression may suggest aggressive disease pathophysiology. Therefore, we extended our analysis to evaluate the relationship between NLRP-3 expression and markers of cellular processes involved in cancer progression (Table 6; Figures 7 and 8). EMT is associated with increased expression of vimentin and matrix metalloproteases (MMPs) and decreased E-Cadherin expression [33]. Among studies reporting EMTmarkers, a significant association between elevated NLRP-3 expression and higher vimentin, MMP expression, and lower E-Cadherin expression in CRC and lung cancers was observed (Table 6) [30,34]. Since the NLRP-3-inflammasome is a potent regulator of immunological response, tumor-immune infiltrate was assessed. Elevated NLRP-3 expression correlated with increased T-cell infiltrate (CD4 and CD8 positive), T-regulatory cells, myeloid-derived suppressor cells (MDSCs), tumor-associated macrophages (TAM), and immune checkpoint markers (PD-1: Programmed Death-1 (PD-1) (Table 6) [26,35].

**Table 6.** Summarized the relationship between NLRP-3 expressions with the markers of cellular processes.

Author/year	Marker of Cellular processes	Primary Outcome
Yang/2017 [30]	EMT	Vimentin expression was significantly associated with NLRP-3 expression in metastatic lung cancer patients (*p* = 0.027).
Marandi/2020 [32]		Higher Vimentin (*p* = 0.019) and MMP9 (*p* = 0.018) expression were linked with higher NLRP-3 expression, while E-cadherin was inversely associated (*p* = 0.001) with CRC.
Chen [2]/2018 (41)	Immune cells recruitment and immune suppression	NLRP-3 expression was significantly associated with T cells (CD4 (*p* < 0.001, CD8 (*p* < 0.01), and Foxp3 (*p* < 0.001), MDSCs (CD33 (*p* < 0.001), CD11b (*p* < 0.001), TAM (CD163 (*p* < 0.01), CD68 (*p* < 0.001), and immune checkpoint markers (PD-1 (*p* < 0.05) and Tim3 (*p* < 0.001) in HNSC cancer patients.
Chen/2022 [26]		A positive association was observed between NLRP-3 and Macrophage type 2 markers CD163 (*p* < 0.042) and CD206 (*p* < 0.0012). Higher expression of both M2-type markers and NLRP-3 contributed to the inferior survival of HNSC patients.

NLRP-3: NOD-like receptor protein 3, MMP-9: Matrix metalloproteinase; E cadherin: epithelial cadherin, MDSCs: myeloid-derived suppressor cell, TAM: tumor-associated macrophages, PD-1: Programmed death-1; Tim3: T cell immunoglobulin mucin-3, FOXP3: forkhead box protein, ALDH1: aldehyde dehydrogenase1, CD44: cluster of differentiation 44, CRC: colorectal cancer patients, HNSC: head and neck cancer.

To gain a deeper understanding of NLRP-3 in relation to EMT and immune cell markers, a pooled odds ratio analysis was performed. The correlation coefficient (*r* value) was converted into Cohen’s d, and then the odds-ratio equivalent using the Borenstein method [36,37]. Notably, higher expression of NLRP-3 was significantly associated with the viemntin (EMT marker) (pooled OR: 4.42, 95% CI: 2.21–8.84, *p* < 0.0001) (Figure 7A), suggesting a robust role of NLRP-3 in regulating the EMT biological process. A significant association of higher expression of NLRP-3 with CD163 (TAMs marker) (CD163) (pooled OR: 2.24, 95% CI: 1.37–3.67, *p* = 0.001) was also observed (Figure 7B), suggesting that NLRP-3 regulates recruitment of immunosuppressive lineages into the tumour microenvironment.

## Discussion

This meta-analysis investigated the prognostic significance of NLRP-3 in patients with solid cancers, which indicated that higher NLRP-3 expression is associated with poorer OS, particularly among patients with HNSC, CRC, and PDAC. It also revealed that higher NLRP-3 expression was associated with adverse clinicopathological parameters.

Chronic inflammation promotes cancer development, progression, and metastasis [38,39,40]. The tumor microenvironment is regulated by immune cell recruitment and cytokine/chemokine release, which contributes to cancer progression and metastasis [38,41–43]. IL-1β is a potent innate immunological cytokine. Sustained secretion of IL-1β promotes tumourigenesis by altering distinct subsets of immune populations and is associated with adverse outcomes [3,42,43]. Targeting IL-1β in cancer received significant attention after re-analysis of the phase III CANTOS trial, which showed that IL-1β neutralization significantly decreased the risk of cancer development and mortality in a dose-dependent manner [4]. The NLRP-3inflammasome is a major source of biologically active, mature IL-1β [18,34,35,44]. The NLRP-3 inflammasome is required for cellular homeostasis. However, gain-of-function mutations in *NLRP-3* cause human autoinflammatory diseases, including cryopyrin-associated periodic syndromes (CAPS) and skin inflammation [45,46]. Given the link between chronic inflammation and cancer, it is expected that NLRP-3 will play a role in cancer development. Indeed, higher NLRP-3 expression was observed in malignant tissue compared to normal tissue [47] and correlated with inferior clinical outcomes in most cancers [19,20,23,25,48]. Surprisingly, the opposite outcome was observed in hepatocellular carcinoma (HCC) [28]. Two independent studies showed that NLRP-3 expression was significantly higher in adjacent normal tissue than in hepatocellular carcinoma [32] and that lower NLRP-3 expression was associated with advanced clinicopathological features in HCC [28]. Whilst confounding factors, including differing research methods, cancer types, histological subtype, ethnicity, sample size, IHC evaluation method and cut-off must be considered, these two independent studies suggest that NLRP-3 may have a protective role in HCC.

The divergent role of NLRP3 in HCC compared to other cancers may be explained by the fact that HCC develops in the context of chronic inflammation, driven by viral infections, such as hepatitis B and C, and alcohol abuse. HCC commonly arises following fibrosis and cirrhosis [49,50]. During chronic infection and alcohol abuse, the NLRP3 inflammasome pathway is activated, leading to sustained inflammation and injury and contributing to tumor formation [49–51]. Expression of NLRP3 inflammasome proteins was shown to be elevated in chronic hepatitis and cirrhotic tissue, but lower in normal and HCC tumor regions [28], suggesting the role of NLRP3 inflammasome activation in the transition from precancerous to cancerous stages differs. Whilst the role for NLRP3 in cancer is typically associated with the production of bio-active IL1β and IL18, NLRP3 also drives a cell death process called pyroptosis through the regulation of gasdermin-D. Interestingly, hepatic stellate cells are activated *via* pyroptosis, which leads to fibrosis and HCC development [49–51]. Additionally, NLRP3 may be involved in the sex-disparity in HCC. Estrogen activates the NLRP3 inflammasome signalling pathway in HCC. Treatment with 17β-estradiol (E2) significantly increased the NLRP3 inflammasome components through ER*β*/MAPK/ERK pathway [52], leading to pyroptotic cell death and decreased tumor growth [52], providing a possible mechanism for the tumor-suppressive effects of NLRP3 in HCC.

Higher NLRP-3 expression is significantly associated with inferior OS and DFS. Importantly, whilst this analysis primarily relied on studies reporting NLRP-3 protein expression, analysis of publicly available transcriptomic data confirmed the link between high *NLRP-3* expression and poor outcome. Further dissection of the data revealed that high NLRP-3 expression corresponds with significantly higher risk in Asian populations, which was not observed in Caucasians. This is consistent with a report of an A to G substitution in *NLRP-3* (rs10754558 and rs35829419) identified in Chinese patients, which was associated with a higher risk of bladder cancer [53]. Additional *NLRP-3* mutations have been identified in Chinese persons (*NLRP-3*-rs12079994, -rs3806265, and -rs4612666) which were linked to gastric cancer [54]. It is important to note that 10 out of 12 studies included in this meta-analysis were from data on Asian populations. Additional studies on other ethnicities are required to ensure the veracity of this observation.

A link between NLRP-3 expression and patient outcomes according to tumor subtypes was identified. High NLRP-3 expression in HNSCC, CRC, and PDAC showed significantly worse OS, which was not observed in breast cancer [25], and low NLRP-3 expression correlated with worse clinical outcomes in HCC. This is consistent with reports of differing roles for NLRP-3 in specific tumor types [28,32]. High NLRP-3 expression or an *NLRP-3* mutation (Q705K) correlated with significantly worse survival in CRC patients [23,55], whereas no significant impact was observed in breast cancer [25]. This observation requires further exploration by detailed mechanistic studies to define the roles of NLRP-3 in specific tumor types.

Intriguingly, when patients were stratified based on chemotherapy regimen, we observed that elevated NLRP-3 expression correlated with significantly poorer response to chemotherapy compared to those without treatment or who underwent surgical resection. These observations are in line with recent studies showing that the NLRP-3/IL-1β axis mediates resistance to chemotherapeutic agents, including gemcitabine (Gem) [56], 5-fluorouracil (5FU) [20], cisplatin [57], and oxaliplatin [58]. For instance, NLRP-3-/IL-1β activated the Wnt/β-catenin signaling pathway, which regulates tumour progression *via* increasing EMT properties and immune evasion following Gem treatment [56] and oxaliplatin treatment [58] thereby causing resistance to these drugs. Additionally, NLRP3 has been shown to be required for effective wound healing [59], which could contribute to the improved outcomes for surgical patients with elevated NLRP3 expression. A limitation in these mechanistic studies is that they have focused on the immune activities of NLRP3. It is possible that the requirement for NLRP3 in pyroptotic cell death could contribute; however, this needs further investigation.

A potential confounding factor in these analyses is the antibody used to determine NLRP-3 expression. Analysis of studies using antibodies from Abcam or Sigma-Aldrich showed a significantly poorer prognosis compared to studies using antibodies from Proteintech. The heterogeneity was higher in the Proteintech antibody-based studies as compared to Abcam and Santa Cruz (*I*^2^ = 78% vs. 28% vs 0%) (Supplementary Figure 2D). A reason for heterogeneity in Proteintech may be due to different types of cancer, the IHC evaluation method, and the sampling method. The IRS showed higher heterogeneity (*I*^2^ = 78%); however, it was associated with poor OS (Supplementary Figure 2E). This higher heterogeneity in the IRS is due, in part, to different IHC evaluation and cut-offs used to define lower and higher expression groups. A standardized scoring system used in the evaluation of NLRP3 expression in future studies will limit this heterogeneity.

Consistent with the link between NLRP-3 expression and poor outcome, we observed a link between NLRP-3 and advanced disease stage. Higher NLRP-3 expressions was associated with larger primary tumor size (higher T stage), more advanced tumor grade, presence of lymph node metastasis, presence of distant metastasis, venous invasion, and neural invasion. These findings indicated that NLRP-3 may regulate cancer progression and metastasis, which may be linked with the inferior prognosis of patients. This is consistent with previous findings that NLRP-3 regulates cell proliferation, progression, EMT, and metastasis to other organs [60,61]. Macrophages interacting with CRC induced the activation of the NLRP-3 inflammasome, resulting in increased migration capacity in tumour cells [62]. However, when *NLRP-3* was knocked out or pharmacologically inhibited, metastasis to the liver was significantly reduced [62]. A similar outcome was observed in melanoma [63].

The EMT process contributes to migration and invasion [64,65]. EMT is a process in which epithelial cells change identity towards a more invasive and migratory state marked by loss of E-Cadherin and gain of markers including Vimentin, Snail, Slug, Zeb1, Zeb2, and Twist [65,66]. Evidence of an association between NLRP-3 and EMT [30,34] may suggest that NLRP-3 drives EMT and hence cancer progression. This meta-analysis included studies that evaluated the EMT status (through assessment of Vimentin and MMP-9 expression). Despite differences in study design, cancer type, sample size, antibody clone, and measurement method (IHC vs Western blot), both studies reported a consistent association between EMT and NLRP-3 expression [30,34]. Pooled OD ratio analysis also supports this finding by demonstrating the significant association between Vimentin and NLRP-3 across the patient sample (pooled OR: 4.42, 95% CI: 2.21–8.84, *p* < 0.0001) (Figure 7). Together, these studies suggest that NLRP-3 may contribute to tumour progression through EMT. One study performed additional mechanistic correlations and showed that NFκB activation, IL-1β, and TGF-β expressions were significantly associated with mesenchymal markers [34]. However, additional studies with more patients are required to define the mechanism.

NLRP-3 expression or activation plays key roles in tumour-immune interactions and has been associated with immune cell recruitment (TAMs, MDSCs, and regulatory T cells (Tregs)) and the suppression of anti-tumour immunity through PD-L1 expression [44,67–69]. In diffuse large B-cell lymphoma (DLBCL), high NLRP-3 expression correlated with PD-L1 expression in tumour tissue [69]. This was directly attributed to NLRP-3 activity because treatment of mouse lymphoma models with the NLRP-3 inhibitor MCC-950 reduced PD-L1 expression [69]. This was associated with an increased percentage of MDSCs, Tregs, and exhausted T-cells (PD-1/TIM-3 double positive) in the peripheral blood of DLBCL patients [69]. Similarly, in two studies on HNSC, NLRP-3 expression positively correlated with PD-1, MDSC, TReg, and TAM markers [26,35]. NLRP-3 expression is not only associated with immune-suppressive and exhausted lineages. A positive correlation between NLRP-3 expression and the abundance of CD4 and CD8 cells was observed in HNSC [35].

The functional importance of NLRP-3-mediated EMT and immune suppression has been studied in CRC, PDAC, and HNSC. In CRC, NLRP-3 was associated with elevated Vimentin expression and reduced E-cadherin expression, which was attributed to the S6K1–GLI1 signaling pathway [70]. This EMT shift promoted immune invasion and metastasis [62]. In PDAC, NLRP3 expression in macrophages drives the polarisation of macrophages 1 (M1/M2b markers) into macrophages 2 (M2a/c/d markers), resulting in increased invasion and migration of PDAC cells [71] and controlling the CD4^+^ T cell differentiation towards an immunosuppressive phenotype [72]. In HNSC, the NLRP-3/IL-1β/HIF-1α signaling axis [26,35,73] augmented the expression of EMT-associated transcription factors, including Twist and Snail, resulting in increased migration and metastasis [73] concomitant with recruitment of immunosuppressive populations including MDSCs, Tregs, and TAMs, collectively enabling tumor progression [35], and decreasing overall survival in HNSCC patients [26].

Taken together, these findings indicate that NLRP-3 is a viable therapeutic target. This is further supported by results from the CANTOS clinical trial, which showed that IL-1β neutralization in the prophylactic setting blocked the initiation of lung cancers [74]. However, in the subsequent CANOPY studies, IL-1β neutralization in patients with existing disease failed [74]. Additionally, whilst many tumour indications showed a correlation between NLRP-3 expression and poor patient outcome, HCC showed an opposing trend. Therefore, it will be important to perform additional studies on prospectively collected samples representing diverse tumour indications and ethnicities. It would also be important to include patient tissue collections throughout disease progression to define the window in tumour development in which targeting NLRP-3 will be most effective.

## Limitation

1. Most studies in this meta-analysis were from Asian patients, particularly Chinese patients. Additional studies of NLRP-3 expression in non-Asian populations are required to clearly define the relationship between NLRP-3, disease and ethnicity.
2. Three studies did not provide HR values. Therefore, we calculated the HR value through Kaplan–Meier curves.
3. In most of the studies included, different antibodies and sampling methods were used to evaluate NLRP-3 (low vs. high) expression.
4. All studies analyzed were retrospective and observational. Additional prospective collections to balance ethnicity and tumour indication are warranted in future analysis.

## Conclusion

Our results indicate that NLRP-3 overexpression is associated with poorer outcomes in many solid tumours and may also be linked to EMT, advanced clinical stage parameters, and metastatic features. However, further mechanistic study is required.

## Supplementary Material

Supplementary data .docx

## Data Availability

All the data produced or analyzed in this meta-analysis are included in this published article and its supplementary files. All data will be made available upon reasonable request.
