# Supplementary material for: Prognostic significance of NLRP-3 expression in solid cancers: a systematic review and meta-analysis
Source: Ann Med. 2026 Aug 20;58(1):2712701. doi: 10.1080/07853890.2026.2712701 (PMC13501775; doi:10.1080/07853890.2026.2712701)
Supplement: Supplementary file 1 [file IANN_A_2712701_SM3779.docx]

**Prognostic significance of NLRP-3 expression in solid cancers: A systematic review and meta-analysis.**

**Supplementary Table 1. Patients’ median follow-up, treatment regimens, and tissue procurement methods of the study cohort.**

| **Author/Year** | **Follow-up**  **(Median months)** | **Chemotherapy regimen** | **Tissue sampling method** |
| --- | --- | --- | --- |
| Feng/2017 (1) | NA | DCF | WTS |
| Xue/2019 (2) | NA | No treatment | TMA |
| He/2020 (3) | NA | No treatment | TMA |
| Shao/2020 (4) | NA | No treatment | WTS |
| Wang/2020 (5) | NA | No treatment | WTS |
| Huang/2021 (6) | NA | Response to chemotherapy | WTS |
| Saponaro/2021 (7) | 67 | NA | TMA |
| Shi/2021 (8) | NA | NA | TMA |
| Chen /2022 (9) | NA | NA | WTS |
| Zheng/2022 (10) | NA | No treatment | WTS |
| Chan Ha/2024 (11) | 53 | NA | TMA |
| Fraile-Martinez/2024(12) | 60 | NA | WTS |

**Supplementary Table 2. NLRP3 antibody details, IHC cut off and immunohistochemical staining method for the NLRP-3 in the included studies**

| **Author/year** | **IHC evaluation method** | **NLRP-3Antibody** | | | | | **Pathologist involvement** | **IHC**  **Cut-off** | **High  NLRP-3 (%)** |
| --- | --- | --- | --- | --- | --- | --- | --- | --- | --- |
|  |  | **Company** | **Source** | **Catalogue number** | **Clone** | **Dilution** |  |  |  |
| Feng/2017 (1) | IRS | Sigma-Aldrich | Rabbit | HPA012878 | Polyclonal | 1:400 | 2 | Low expression: 0–6  High expression: 7–12 | 71 (68.9) |
| Xue/2019 (2) | IRS | Abcam | Rabbit | ab214185 | Polyclonal | 1:200 | NA | Low expression: 0–6  High expression:7–12 | 49 (47.1) |
| He/2020 (3) | NA | Protein tech | Rabbit | 19771-1-AP | Polyclonal | NA | 2 | NA | 59 (40.97) |
| Shao/2020 (4) | NA | Abcam | Rabbit | ab214185 | Polyclonal | 1:1000 | 2 | NA | 29 (48.33) |
| Wang/2020 (5) | IRS | Abclonal | Rabbit | A5652 | Polyclonal | 1:100 | NA | Low expression: 0-4  High expression:5–12 | 56 (41.48) |
| Huang/2021(6) | IRS | Affinity | Rabbit | DF7438 | Polyclonal | NA | 2 | Low expression: 0–2  High expression: 3–9 | 25 (45.4) |
| Saponaro/2021 (7) | ROC | NA | NA | NA | NA | NA | 2 | Low expression: <20%  High expression: >20% | 53 (50%) |
| Shi/2021 (8) | IRS | Protein tech | Rabbit | 19771-  1-AP | Polyclonal | NA | NA | Low expression: 0-4  High expression: 5-9 | 67 (67) |
| Chen /2022 (9) | IRS | Sigma-Aldrich | NA | NA | NA | 1:200 | 2 | Low expression 0,1  High expression: 2,3 | 39 (37.5) |
| Zheng/2022 (10) | IRS | Santa Cruz | Rabbit | SC-66846 | Polyclonal | 1:250 | 2 | Low expression: ≥4  High expression: <4 | 56 (57.1) |
| Chan Ha/2024 (11) | IRS | Adipogen | Mouse | 20B-0014-C100, | Monoclonal | 1:150 | 2 | Low expression 0,1  High expression: 2,3 | 235 (66.9) |
| Fraile-Martinez/2024 (12) | IRS | Abcam | Rabbit | ab214185 | Polyclonal | 1:250 | 2 | Low expression 0,1  High expression: 2,3 | 25 (60.9) |

**Abbreviation:** WTS: whole tissue section, TMA: tissue microarray, IRS: immunoreactivity score, NA: not available, ROC: receiver operating characteristic curve, mAb: monoclonal Antibody, pAB: polyclonal antibody.

**Supplementary Table 3. Results of quality assessment using the Newcastle-Ottawa Scale system of the study cohort**

| First author, year | Selection^1^ | | | | Comparability^2^ | Outcome^3^ | | |  |
| --- | --- | --- | --- | --- | --- | --- | --- | --- | --- |
|  | Exposed Cohort ★ | Non-exposed cohort ★ | Ascertainment of exposure ★ | The outcome of the study was not presented at the start of study★ | Control for important factors or additional factors ★★ | Outcome  Assessment ★ | Adequate  follow-up★ | Loss to follow-up★ | Total Score |
| Wei/2014 (13) | ★ | ★ | ★ |  | ★★ |  |  |  | 5 |
| Feng/2017 (1) | ★ | ★ | ★ |  | ★★ | ★ | ★ |  | 7 |
| Yang/2017(14) | ★ | ★ | ★ |  | ★★ |  |  |  | 5 |
| Chen (2)/2018 (15) | ★ | ★ | ★ |  | ★★ |  |  |  | 5 |
| Xue/2019 (2) | ★ | ★ | ★ |  | ★★ | ★ | ★ |  | 7 |
| He/2020 (3) | ★ | ★ | ★ |  | ★★ | ★ | ★ |  | 7 |
| Marandi/2020 (16) | ★ | ★ | ★ |  | ★★ |  |  |  | 5 |
| Shao/2020 (4) | ★ | ★ | ★ |  | ★★ | ★ | ★ |  | 7 |
| Wang/2020 (5) | ★ | ★ | ★ |  | ★★ | ★ | ★ |  | 7 |
| Huang/2021(6) | ★ | ★ | ★ |  | ★★ | ★ | ★ |  | 7 |
| Saponaro/2021 (7) | ★ | ★ | ★ |  | ★★ | ★ | ★ | ★ | 8 |
| Shi/2021 (8) | ★ | ★ | ★ |  | ★★ | ★ | ★ |  | 7 |
| Chen/2022 (9) | ★ | ★ | ★ |  | ★★ | ★ | ★ |  | 7 |
| Zheng/2022 (10) | ★ | ★ | ★ |  | ★★ | ★ | ★ |  | 7 |
| Chan Ha/2024 (11) | ★ | ★ | ★ |  | ★★ | ★ | ★ | ★ | 8 |
| Fraile-Martinez/2024(12) | ★ | ★ | ★ |  |  | ★ | ★ | ★ | 6 |

1“Selection” part consists of the representativeness of cases, selection of controls, exposure ascertainment, and no death when the study started.

2“Comparability” contains a comparison between each group and additional factors comparable on confounders.

3“Outcome” includes outcome assessment, adequate follow-up, and loss to follow-up rate.

**Supplementary Table 4. Outcome of the risk of bias of the study cohort.**

| **Studies** | **Study participation** | **Study attrition** | **Prognostic factor measurement** | **Outcome assessment** | **Study confounding** | **Statistical analysis and reporting** | **Overall Score** |
| --- | --- | --- | --- | --- | --- | --- | --- |
| Wei/2014 (13) | Low | High | Low | High | High | Low | High |
| Feng/2017 (1) | Moderate | High | Low | Low | Low | Low | Moderate |
| Yang/2017(14) | Moderate | High | Low | High | High | Low | High |
| Chen (2)/2018 (15) | Moderate | High | Moderate | High | High | Low | High |
| Xue/2019 (2) | Moderate | Low | Moderate | Low | Low | Low | Moderate |
| He/2020 (3) | Moderate | High | Moderate | Low | Low | Low | Moderate |
| Marandi/2020 (16) | Moderate | High | Moderate | High | High | Low | High |
| Shao/2020 (4) | Moderate | Low | Moderate | Low | High | Low | Moderate |
| Wang/2020 (5) | Moderate | Low | Low | Low | Low | Low | Low |
| Huang/2021(6) | Moderate | High | Low | Low | Moderate | Low | Moderate |
| Saponaro/2021 (7) | Moderate | Low | Low | Low | High | Low | Moderate |
| Shi/2021 (8) | Low | Low | Moderate | Low | Low | Low | Low |
| Chen/2022 (9) | Moderate | Low | Low | Low | High | Low | Moderate |
| Zheng/2022 (10) | Moderate | High | Low | Low | High | Low | High |
| Chan Ha/2024 (11) | High | Low | Low | Low | Low | Low | Moderate |
| Fraile-Martinez/2024(12) | High | Low | Low | Low | High | Low | High |

**Supplementary Table 5. NOS and QUIPS overall scores evaluated by both reviewers, and the final consensus**

|  | **NOS Score** | | |  | **QUIPS Score** | | |
| --- | --- | --- | --- | --- | --- | --- | --- |
| **Studies** | **Reviewer 1** | **Reviewer 2** | **Overall Score** | **Category** | **Reviewer 1** | **Reviewer 2** | **Overall Score** |
| Wei/2014 (13) | 5 | 5 | 5 | Low | High | High | High |
| Feng/2017 (1) | 7 | 7 | 7 | High | Low | Low | Moderate |
| Yang/2017(14) | 5 | 5 | 5 | Low | High | High | High |
| Chen (2)/2018 (15) | 5 | 5 | 5 | Low | High | High | High |
| Xue/2019 (2) | 7 | 7 | 7 | High | Low | Low | Moderate |
| He/2020 (3) | 7 | 7 | 7 | High | Low | Low | Moderate |
| Marandi/2020 (16) | 5 | 5 | 5 | Low | High | High | High |
| Shao/2020 (4) | 7 | 7 | 7 | High | Low | High | Moderate |
| Wang/2020 (5) | 7 | 7 | 7 | High | Low | Low | Low |
| Huang/2021(6) | 7 | 7 | 7 | High | Low | Moderate | Moderate |
| Saponaro/2021 (7) | 8 | 8 | 8 | High | Low | High | Moderate |
| Shi/2021 (8) | 7 | 7 | 7 | High | Low | Low | Low |
| Chen/2022 (9) | 7 | 7 | 7 | High | Low | High | Moderate |
| Zheng/2022 (10) | 7 | 7 | 7 | High | Low | High | High |
| Chan Ha/2024 (11) | 8 | 8 | 8 | High | Low | Low | Moderate |
| Fraile-Martinez/2024(12) | 6 | 6 | 6 | Low | Low | High | High |

**Supplementary Table 6. Grades of Recommendation, Assessment, Development, and Evaluation (GRADE) summary of the included study.**

| **Outcome** | **Number of studies/**  **participants** | **Risk of Bias from QUIPS** | **Pooled Result (hazard ratio and 95%CI)** | **Certainty of evidence (GRADE)** | **Reasons for downgrading** |
| --- | --- | --- | --- | --- | --- |
| Overall survival | 12 (1,731) | Downgrade 1 level | 2.12 [1.49, 3.03] | Very low | Risk of bias, inconsistency, and publication bias |
| Disease-Free Survival | 4 (583) | Downgrade 1 level | 1.86 [1.30, 2.65] | Very low | Risk of bias, indirectness, and imprecision |

**Supplementary Figure 1.** Forest plot showing the relationship between NLRP-3 expression and overall survival (OS) (A) ethnicity, (B) treatment regimen, (C) cancer types, and (D) analysis types. HR and the corresponding 95% confidence interval (CI) were also reported for each study. A hazard ratio of more than 1 is associated with a poor prognosis, and below 1 is considered a good prognosis.

**Supplementary Figure 2.** Forest plot showing the relationship between NLRP-3 expression and overall survival (OS) (A) sample size, (B) sampling method, (C) source of HR extraction, (D) antibody types, (E) antibody clones, and (F) IHC scoring methods. HR and the corresponding 95% confidence interval (CI) were also reported for each study. A hazard ratio of more than 1 is associated with a poor prognosis, and a ratio below 1 is considered a good prognosis.

**Supplementary Figure 3.** Meta-regression plot demonstrates the correlation between (A) antibody clones, (B) IHC evaluation methods, (C) sampling methods and NLRP-3 expression in solid cancer. **Abbreviation:** mAb: monoclonal Antibody, pAB: polyclonal antibody, WTS: whole tissue section, TMA: tissue microarray, IRS: immunoreactivity score, ROC: receiver operating characteristic curve

**Supplementary Figure 4.** Forest plot showing the relationship between NLRP-3 expression and various clinicopathological parameters (A) age, (B) gender, (C) tumour size, and (D) T stage. The odds ratio, along with the corresponding 95% confidence interval (CI) was also reported for each study.

**Supplementary Figure 5**. Forest plot showing the relationship between NLRP-3 expression and various clinicopathological parameters (A) N stage, (B) M stage, (C) TNM stage, and (E) tumour grade. The odds ratio with the corresponding 95% confidence interval (CI) was also reported for each study.

**Supplementary Figure 6**. Forest plot showing the relationship between NLRP-3 expression and various clinicopathological parameters (A) venous invasion, (B) neural invasion, (C) smoking, and (D) alcohol. The odds ratio with the corresponding 95% confidence interval (CI) was also reported for each study.

**References**

1.	Feng X, Luo Q, Zhang H, Wang H, Chen W, Meng G, et al. The role of NLRP3 inflammasome in 5-fluorouracil resistance of oral squamous cell carcinoma. Journal of Experimental & Clinical Cancer Research. 2017;36(1):1-14.

2.	Xue Y, Du H-D, Tang D, Zhang D, Zhou J, Zhai C-W, et al. Correlation between the NLRP3 inflammasome and the prognosis of patients with LSCC. Frontiers in oncology. 2019;9:588.

3.	He Z-F, Jin X-R, Lin J-J, Zhang X, Liu Y, Xu H-L, et al. NALP3 orchestrates cellular bioenergetics to facilitate non-small cell lung cancer cell growth. Life sciences. 2020;241:117165.

4.	Shao X, Lei Z, Zhou C. NLRP3 promotes colorectal cancer cell proliferation and metastasis via regulating epithelial mesenchymal transformation. Anti-Cancer Agents in Medicinal Chemistry (Formerly Current Medicinal Chemistry-Anti-Cancer Agents). 2020;20(7):820-7.

5.	Wang B, Li H, Wang X, Zhu X. The association of aberrant expression of NLRP3 and p-S6K1 in colorectal cancer. Pathology-Research and Practice. 2020;216(1):152737.

6.	Huang Z, Chen H, Wang S, Wei H, Wang X, Shen R, et al. NLRP3 Overexpression Associated With Poor Prognosis and Presented as an Effective Therapeutic Target in Osteosarcoma. Frontiers in Pharmacology. 2021;12:724923.

7.	Saponaro C, Scarpi E, Sonnessa M, Cioffi A, Buccino F, Giotta F, et al. Prognostic value of NLRP3 inflammasome and TLR4 expression in breast cancer patients. Frontiers in oncology. 2021:3502.

8.	Shi F, Wei B, Lan T, Xiao Y, Quan X, Chen J, et al. Low NLRP3 expression predicts a better prognosis of colorectal cancer. Bioscience Reports. 2021;41(4).

9.	Chen L, Wan SC, Mao L, Huang CF, Bu LL, Sun ZJ. NLRP3 in tumor-associated macrophages predicts a poor prognosis and promotes tumor growth in head and neck squamous cell carcinoma. Cancer Immunol Immunother. 2023;72(6):1647-60.

10.	Zheng L, Liu H. Prognostic association between NLRP3 inflammasome expression level and operable pancreatic adenocarcinoma. The International Journal of Biological Markers. 2022:03936155221096690.

11.	Ha S-C, Park YS, Kim J. Prognostic significance of pyroptosis-associated molecules in endometrial cancer: a comprehensive immunohistochemical analysis. Frontiers in Oncology. 2024;14.

12.	Fraile-Martinez O, García-Montero C, Pekarek L, Saz JV, Álvarez-Mon M, Barrena-Blázquez S, et al. Decreased survival in patients with pancreatic cancer may be associated with an increase in histopathological expression of inflammasome marker NLRP3. Histol Histopathol. 2024;39(1):35-40.

13.	Wei Q, Mu K, Li T, Zhang Y, Yang Z, Jia X, et al. Deregulation of the NLRP3 inflammasome in hepatic parenchymal cells during liver cancer progression. Laboratory Investigation. 2014;94(1):52-62.

14.	Yang D, Cao X, Wang F, Jiang H, Feng D, Guo H, et al. LFG-500, a novel synthetic flavonoid, suppresses epithelial–mesenchymal transition in human lung adenocarcinoma cells by inhibiting NLRP3 in inflammatory microenvironment. Cancer Letters. 2017;400:137-48.

15.	Chen L, Huang CF, Li YC, Deng WW, Mao L, Wu L, et al. Blockage of the NLRP3 inflammasome by MCC950 improves anti-tumor immune responses in head and neck squamous cell carcinoma. Cell Mol Life Sci. 2018;75(11):2045-58.

16.	Marandi Y, Hashemzade S, Tayebinia H, Karimi J, Zamani A, Khodadadi I. NLRP3-inflammasome activation is associated with epithelial-mesenchymal transition and progression of colorectal cancer. Iran J Basic Med Sci. 2021;24(4):483-92.
